# Lipiodol retention pattern assessed by cone beam computed tomography during conventional transarterial chemoembolization of hepatocellular carcinoma: accuracy and correlation with response

**DOI:** 10.1186/s40644-016-0090-4

**Published:** 2016-10-03

**Authors:** Jungang Hu, Majid Maybody, Guang Cao, Xiao Wang, Hui Chen, Xu Zhu, Renjie Yang, Xiaodong Wang

**Affiliations:** 1 Department of Interventional Radiology, Peking University Cancer Hospital & Institute, Key laboratory of Carcinogenesis and Translational Research (Ministry of Education), Beijing, 100142 China; 2 Department of Radiology, Interventional Radiology Service, Memorial Sloan-Kettering Cancer Center, 1275 York Ave, New York, 10021 NY USA; 3 Department of Epidemiology and Biostatistics, Peking University Sixth Hospital, Beijing, 100191 China

**Keywords:** Cone beam computed tomography, Fluoroscopy, Transarterial chemoembolization, Hepatocellular carcinoma, Liver

## Abstract

**Background:**

To investigate accuracy of intraprocedural cone beam computed tomography (CBCT) compared to fluoroscopy for detection of lipiodol retention pattern during conventional transarterial chemoembolization (cTACE) of hepatocellular carcinoma (HCC) and its correlation with short-term response.

**Methods:**

Between September 2013 and July 2014, 29 patients with HCC underwent chemoembolization of 51 tumors (mean diameter 28.1 mm, range 10.0–136.3 mm). Lipiodol retention pattern was assessed by CBCT at the endpoint of cTACE compared by fluoroscopy. Depending on the pattern of tumor covered by lipiodol three classes were defined: complete (more than 90 %, no peripheral defects), moderate (50–90 %, some with or without peripheral defects), and poor (less than 50 %). Tumor response was assessed by modified Response Evaluation Criteria in Solid Tumors (mRECIST) based on follow-up contrast enhanced (CE) computed tomography (CT) or magnetic resonance imaging (MRI) obtained 4–6 weeks post-cTACE. Correlations between lipiodol retention patterns on CBCT and fluoroscopy as well as tumor response were assessed using multivariate logistic regression.

**Results:**

Of 51 hepatic tumors, 40 (78.4 %) had complete response (CR); 8 (15.7 %) had partial response (PR); 1 (2.0 %) had stable disease (SD); and 2 (3.9 %) had progressive disease (PD). The degree of lipiodol retention scored excellent, moderate, and poor, in fluoroscopic images vs CBCT images were 23 (45.1 %) vs 39 (76.5 %), 19 (37.3 %) vs 11 (21.6 %), and 9 (17.6 %) vs 1 (2.0 %), respectively. Lipiodol retention assessment with CBCT (A_z_ = 0.75) is more accurate than fluoroscopy (A_z_ = 0.54) in predicting target tumor response. Other than lipiodol retention pattern assessed with CBCT (*p* = 0.01), tumor size (*p* = 0.04) is an independent predictors of CR.

**Conclusion:**

CBCT is more accurate than fluoroscopy in classification of lipiodol retention pattern in HCC tumors at the time of cTACE. CBCT could be used as a reliable intra precedural monitoring modality of cTACE.

## Background

Hepatocellular carcinoma (HCC) is the sixth most common cancer and the third leading cause of cancer-related-death worldwide [1]. Curative treatments including resection, liver transplantation and local ablation are indicated in less than 30 % of patients at the time of diagnosis [2]. Transarterial chemoembolization (TACE) is the current standard of care for HCC patients with unresectable intermediate-stage disease and has been reported to prolong survival [3–5].

Early assessment of the effectiveness of TACE and identifying predictors of tumor response are crucial for successful management. Intraprocedural image monitoring is important to assess the endpoint of TACE and application of additional treatment if needed while the patient is still in the angiography suite.

cTACE uses iodized oil (lipiodol) (Guerbet, Aulnay-sous-Bois, France) as a carrier of chemotherapeutic agents [6, 7] and the degree of intratumoral lipiodol retention has been shown to correlate with tumor necrosis and local tumor recurrence with HCC [8, 9].

Visualizing distribution of lipiodol after chemoembolization by fluoroscopy or computed tomography (CT) scan ensures tumor targeting [10]. However, fluoroscopic imaging may fail to detect lack of lipiodol accumulation within the tumor [11]. Computed tomography is more accurate to depict cross-sectional lipiodol distribution [12], but it is cumbersome to transfer patients from the angiography suite to the CT suite since the hybrid angiography-CT systems are not always available.

Cone beam computed tomography (CBCT) using a flat-panel detector is increasingly used and widely available [13]. It has been shown to have lipiodol detection rate comparable with multidetector CT imaging [14]. Lipiodol retention with the help of three dimensional quantification software was found to correlate with tumor response in HCC in a recently published paper [15]. But this software is not widely available.

The goal of this study was to investigate whether gross lipiodol retention pattern on CBCT imaging immediately after cTACE of hepatocellular carcinoma can be used as predictor of tumor response by mRECIST, and whether this could be used as a reliable intra procedural monitoring modality.

## Methods

### Subjects

Authorization from the Institutional Ethics Committee was not needed for this retrospective study in our center. Informed consent was obtained from each patient. All cTACE interventions performed between September 2013 and July 2014 were reviewed. From the total of 189 patients, the following were excluded: non HCC etiology (*n* = 38), prior cTACE (*n* = 98), other treatments within 4 weeks prior to cTACE (*n* = 3), lack of CBCT during cTACE (*n* = 12), significant image artifacts on CBCT (*n* = 3), poor quality follow-up CT or MRI imaging (*n* = 2) and those lost to follow-up (*n* = 4). The study group included all patients (*n* = 29) with HCC who were eligible to undergo their first cTACE as described below, whose cTACE included CBCT, who had not undergone systemic therapy within one month prior to cTACE until the first follow-up imaging study 4–6 weeks after cTACE, who had undergone dynamic contrast-enhanced MR or CT imaging within one month before cTACE and had follow up imaging 4–6 weeks after cTACE.

The diagnosis of HCC was confirmed by biopsy or by characteristic radiologic findings for tumors larger than one cm in patients at risk for HCC such as cirrhotics or hepatitis B carriers. Eligibility criteria for cTACE were as follows: Eastern Cooperative Oncology Group (ECOG) performance status ≤2; Child-Pugh classification A or B; tumor involvement less than 60 % of total liver volume; absence of portal vein tumor thrombus; absence of ascites; albumin > 2.5 g/dl; alanine aminotransferase and aspartate aminotransferase <5 times the upper normal limit; total serum bilirubin <3.0 mg/dl; serum creatinine <2.0 mg/dl; platelet count >50,000/mm^3^ and international normalized ratio (INR) ≤1.5. Patient demographics and tumor data are listed in Table 1.

**Table 1:** Baseline characteristics of patients with HCC

Characteristic	No. of Patient/Mean
No. of patients	29
Age (y)	59.2 ± 11.6
Sex (male/female)	26/3
Etiology	
Hepatitis B	27 (93.1 %)
Hepatitis C	1 (3.4 %)
ECOG performance status (0/1/2/3/4)	21/7/1/0/0
Status 0	21 (72.4 %)
Status 1	7 (24.1 %)
Status 2	1 (3.5 %)
Child-Pugh class (A/B/C)	26/3/0
A	26
B	3
AFP (ng/ml)	
< 10	5 (17.2 %)
10-400	15 (51.7 %)
> 400	9 (31.0 %)
Prior hepatic resection	
Yes	7 (24.1 %)
No	22 (75.9 %)
Index tumor numbers (per patient)	3.22 ± 2.20 (1–5)
Size mean + sd (range, in mm)	28.1 ± 24.4 (10.0-136.3)
Tumor pattern	
Unifocal	12
Multifocal	39
Tumor location	
Caudal lobe	1
Left lobe	14
Right lobe	35
Border	1

### Transarterial chemoembolization

All cTACE were performed using a 40 cm flat panel angiography system (Innova 4100, GE Healthcare, Waukesha, WI, USA). The technique for embolization has been previously described [4, 16]. Details of the hepatic artery anatomy, feeding artery and location of tumors, and portal vein patency and flow direction were obtained from hepatic and superior mesenteric arteriography via a 5 Fr catheter. A 2.7 to 2.8 Fr microcatheter (Progreat, Terumo, Japan) was used to selectively cannulate tumor feeders. In cases where there were multifocal lesions in one lobe, the micro-catheter was positioned proximally in the feeding artery of a sector or a lobe. The emulsion consisted of 5–30 ml of lipiodol (Guerbet, Aulnay-sous-Bois, France) mixed with 40–60 ml Epirubicin (Hisun Pharmaceutical, Zhejiang, China), which was slowly injected through microcatheter under fluoroscopic-monitoring. The diameter of the index lesion in cm was multiplied by two to calculate the volume of iodized oil in ml used to make the emulsion. The entire emulsion was injected unless stasis was achieved or the portal vein around the tumor was visualized. This was followed with injection of particles such as 150–350 and 350–550 μm gelfoam particles (Alicon Pham SCI &TEC, Hangzhou, China) or 100–300 and 300–500 μm embosphere particles (Biosphere Medical, Rockland, Massachusetts, USA) until stasis was achieved. In cases where the territory of more than one vessel was treated, the emulsion was split between the receiving vessels at the discretion of angiographer. At the end point of cTACE based on fluoroscopy, CBCT was performed to assess the retention pattern of lipiodol.

### Cone beam computed tomography

During a 10 s acquisition, 293 projection images were obtained with 180° rotation (20°/s). Cross-sectional images with 1.8 mm slice thickness and 512 × 512 × 512 matrix size were reconstructed from the projections. Images were reconstructed within approximately two minutes.

### Image analysis

The patterns of lipiodol retention by the tumor (s) on CBCT and fluoroscopy images were evaluated at the workstation (Advantage Workstation 4.3, GE Healthcare). The retention patterns were classified by two experienced radiologists (14, 16 years experiences in interventional radiology) and decisions were reached by consensus. The retention patterns were classified as “complete” (more than 90 % dense retention of the tumor, no peripheral defects), “moderate” (50–90 % dense retention, with or without peripheral defects), and “poor” with less than 50 % dense retention of the tumor, with peripheral defects or no retention at all.

### Tumor response evaluation

All study patients were evaluated by baseline CE-CT or CE-MRI within 1 month before cTACE. The index tumors were identified and baseline measurements made. The follow-up CE-CT or CE-MRI scan was performed 4–6 weeks after cTACE. Tumor response was assessed on the follow-up images according to the mRECIST criteria [17]. Unidimensional measurement of the longest diameter was recorded for each index tumor, which was selected according to mRECIST standards. Tumor responses were evaluated by a third experienced radiologist (16 years experiences in abdominal imaging) who was blinded to the study data separately.

Response was defined as follows: “complete response” (CR) as disappearance of all intratumoral enhancement; “partial response” (PR) as < 30 % decrease in diameters of enhancing tumor from baseline; “progressive disease” (PD) as > 20 % increase in diameters of enhancing tumor from baseline; and “stable disease” (SD) as all other tumors.

### Statistical analysis

The patient demographics and tumor data were recorded in a secure database and expressed as mean and median. To assess risk factors for tumor response, univariate and multivariate analyses were performed through binary logistic regression. All analyses were performed by an independent investigator using SPSS (version 18.0). P value of less than 0.05 was set as significant for all analyses.

## Results and discussion

### Lipiodol retention pattern and tumor response

**Fig. 1:**
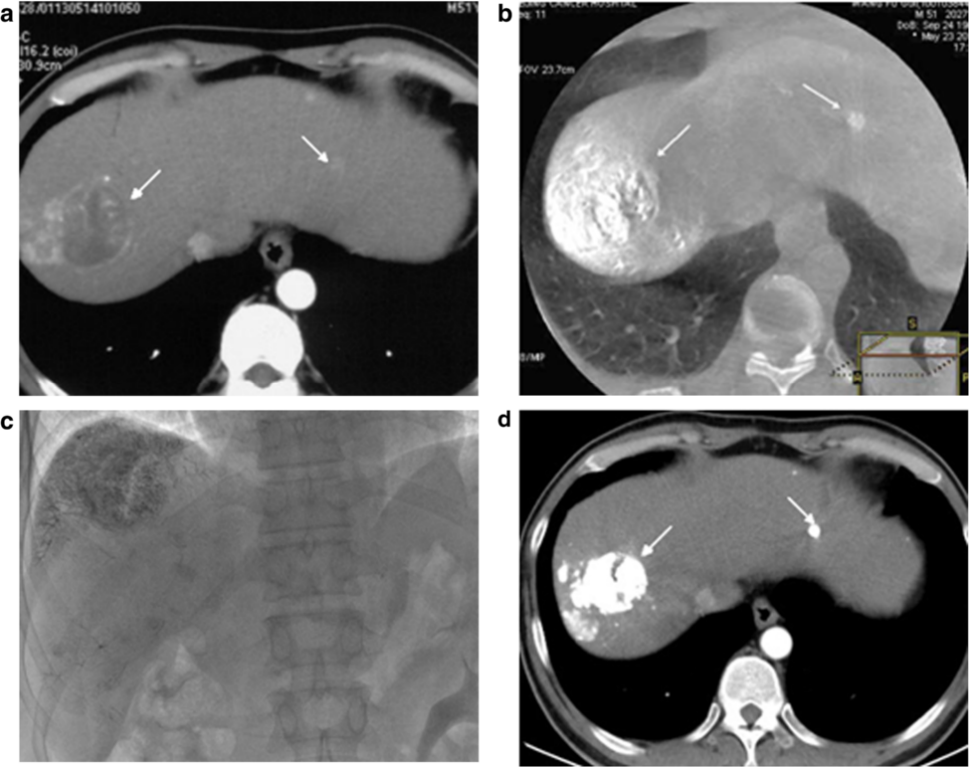
A 52-year-old male with multiple HCCs. **a** Preprocedure arterial phase contrast-enhanced CT shows index tumors in segments II and VII (*arrows*); **b** CBCT immediately after TACE showed these two tumor with complete lipiodol retention pattern concordance with CE-CT (*arrows*); **c** On the fluoroscopy image, complete lipiodol retention pattern is well shown on the large tumor in segment VII, but no retention in the small tumor in segment II. **d** Postprocedure 1 month follow-up contrast-enhancement CT shows both tumors without contrast enhancement (*arrows*) representing complete response

**Fig. 2:**
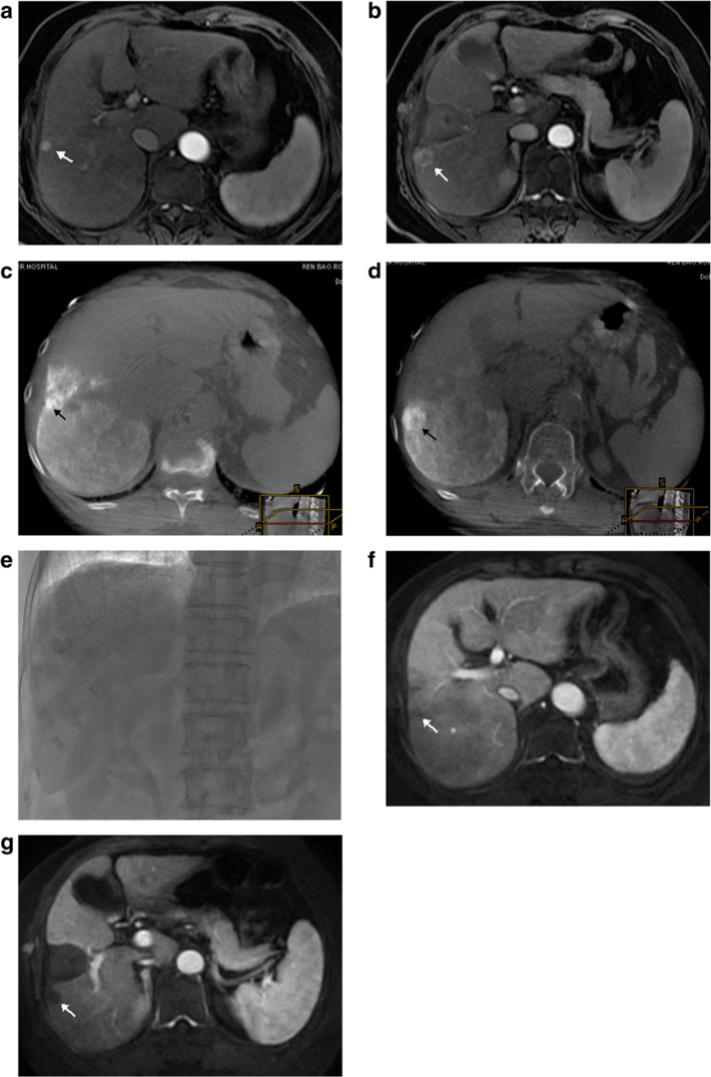
A 56-year-old female with multiple HCCs and prior history of radiofrequency ablation. **a**, **b** Preprocedure arterial phase contrast-enhanced MRI shows two tumors adjacent to the ablation margin in the right lobe (*arrows*); **c**, **d** Cone beam CT immediately after completion of TACE showed these two tumors with complete lipiodol retention pattern without defect concordant with MRI; **e** On the fluoroscopy image, tumor lipiodol retention was not clearly seen. **f**, **g** Postprocedure 1 month follow-up contrast-enhancement MRI showed necrosis without contrast enhancement in both tumors (arrows) representing complete response

The pattern of lipiodol retention was different between fluoroscopy and CBCT (Figs. 1 and 2). In fluoroscopic images of 51 tumors, the complete, moderate and poor classes of lipiodol retention patterns were 23 (45.1 %), 19 (37.3 %), and 9 (17.6 %), respectively. In CBCT images, however, the results were 39 (76.5 %), 11 (21.6 %), and 1 (2.0 %), respectively. Of all the tumors, 78.4 % (*n* = 40) showed a CR, and 15.7 % (*n* = 8), 2.0 % (*n* = 1), and 3.9 % (*n* = 2) showed PR, SD, and PD.

In the “excellent” retention tumors by CBCT, 34/39 tumors (87.2 %) showed CR and 5/39(12.8 %) tumors showed PR. By comparison, in the “excellent” retention tumors by fluoroscopy group, 18/23(78.3 %) showed CR response, while 16/19 (84.2 %) “moderate” retention tumors and 6/9 (66.7 %) “poor” retention tumors still showed CR response. The pattern of lipidol retention by CBCT versus fluoroscopy and the number of tumors which demonstrated completed vs partial response, stable disease, or progression of disease for CBCT versus fluoroscopy are listed in Table 2.

**Table 2:** Lipiodol retention variables and tumor response

Variable		Response		
	PD	SD	PR	CR
Fluoroscopy				
Excellent (23)	0	0	5	18
Moderate (19)	1	0	2	16
Poor (9)	1	1	1	6
Lip-CBCT				
Excellent (39)	0	0	5	34
Moderate (11)	1	1	3	6
Poor (1)	1	0	0	0

### Predictors of response

The accuracy for predicting tumor response by assessing the accumulation of iodized oil shows significant difference between the CBCT (A_z_ = 0.75) and fluoroscopy (A_z_ = 0.54). The correlation between tumor response was stronger with CBCT classification than fluoroscopy classification. Thirty-four among 39 tumors (87.2 %) in the “complete” retention tumors by CBCT showed CR. By comparison, in the classification based on the fluoroscopy, 18 tumors (78.3 %) out of 23 “complete” lipiodol retention class showed CR response.

The correlation of patient, tumor, and lipiodol retention pattern variables and CR by univariate and multivariate analyses are reported in Tables 3 and 4. Multivariate analysis suggests that lipiodol retention pattern by CBCT and tumor size are independent predictors of achieving a complete radiologic response (OR 19.17, *p* = 0.01 and OR 0.88, *p* = 0.04 respectively).

**Table 3:** Univariate analysis of predictors of CR

Factors	OR	95 % CI	*p*
Patients and tumor variables			
Sex (male/female)	0.73	(0.51,1.04)	0.01
Age(y) ≥ 60 vs. <60	2.07	(0.46,9.40)	0.48
Size ≥ 30 mm vs. <30 mm	0.32	(0.08,1.32)	0.35
Location Left vs. Right	1.01	(0.25,4.12)	1.00
Unifocal vs. Multifocal	0.32	(0.04,2.88)	0.29
Child-Pugh A vs. B	0.83	(0.17,4.01)	0.82
AFP (≥ 400 vs. < 400)	4.07	(0.46,35.75)	0.25
Prior hepatic resection Yes vs. No	0.57	(1.02,1.30)	1.15
Lipiodol retention on Fluoroscopy Excellent vs. Moderate and Poor	0.76	(0.22,2.64)	0.75
Lipiodol retention on CBCT Excellent vs. Moderate and Poor	5.17	(1.13,23.55)	0.03
Etiology Hepatitis B virus vs. negative	2.19	(0.56,8.61)	0.27

**Table 4:** Multivariate models of predictors of CR

Model	OR	95 % CI	*p*
Tumor Size	0.88	(0.01,0.90)	0.04
Lipiodol retention in Lip-CBCT Excellent vs. Moderate and Poor	19.17	(1.87,196.49)	0.01

## Discussion

Earlier determination of the tumor response after cTACE is essential in decision making for application of additional treatments in patients with HCC. Histopathologic examination for the determination of tumor response after transarterial chemoembolization is neither feasible nor acceptable. Imaging techniques, such as 4–6 weeks follow-up contrast-enhanced CT and MR imaging are widely used to evaluate the therapeutic effect [17, 18]. For both EASL and mRECIST criteria, measurement of enhanced tumor portion rather than the total visible tumor size has been used to evaluate tumor response in HCC patients after cTACE [17, 19]. Based on this post-procedure assessment imaging, a repeat cTACE or tumor ablation would be needed in case there was residual viable tumors were identified.

Tumor progression for the incompletely treated tumors could be expected during this time interval. Given this risk, some investigators have reported the contrast-enhanced ultrasonography (US) performed at least two and seven days after cTACE could be similar to the dynamic follow-up CT and be predictive of tumor outcome [20]. Some clinical studies have shown the ability of diffusion-weighted MR imaging to help quantify tumor necrosis after transcatheter liver-directed therapy [21, 22]. However, MR or US imaging are separate tests and require logistics for scheduling. Intraprocedural image monitoring is important to determine the endpoint of cTACE and application of additional treatment if needed while in the angiography suite. Utilization of CBCT performed at the time of cTACE for prediction of tumor response obviates the need for additional testing.

CBCT with flat-panel detector acquired during the cTACE procedure has been shown to have several advantages such as identifying the tumor feeding arteries, occult lesions, cyctic artery, etc. [23, 24]. Loffroy et al. showed dual-phase CBCT can be used to predict the short term response to TACE with drug-eluting beads [25].

cTACE with lipiodol is widely used and has comparable efficacy to DEB-TACE [26]. Lipiodol not only acts as a carrier of chemotherapeutic agents, but its use has yielded fairly beneficial therapeutic results. It exposes the tumor to high concentrations of the chemotherapeutic agents for a prolonged period of time, while minimizing systemic toxicity [27]. In this study we demonstrated the lipiodol retention pattern on CBCT immediately after cTACE can be used to predict the short-term tumor response at 4–6 weeks follow-up in HCC patient by mRECIST.

Lipiodol retention pattern after cTACE on follow-up CT imaging has been shown to be a prognostic marker [8, 18, 28]. Multidetector computed tomography (MDCT) can evaluate lipiodol retention after cTACE [14], however, the exact method for assessing lipiodol retention is to perform the post-procedural CT directly after cTACE and the patient must be transferred from the angiography suite to the CT scanner. Despite advances in the angiography unit, fluoroscopic imaging may not accurately show lipiodol retention pattern in the tumor because it cannot provide volumetric information such as CBCT [11]. A combined CT-angiography system with a stand alone CT scanner is useful to evaluate the lipiodol retention pattern; however, the system is expensive and requires a large room [9].

In this study, we have demonstrated that CBCT imaging performed during cTACE is superior to fluoroscopic imaging for assessing the lipiodol retention pattern in HCC tumors. A theoretical downside may be that CBCT may be less sensitive given its inferior spatial and contrast resolution compared with conventional CT [29]. However, it offers the advantage of imaging during a cTACE procedure without the necessity of transferring the patient. In one study, Rongxin Chen et al. have reported that CBCT imaging has a similar capability to assess Lipiodol retention as MDCT [14]. Correlation between contrast retention pattern in liver tumors detected on intraprocedural CT images during transcatheter bland embolization and tumor response has been reported [30]. Strong correlation between lipiodol retention on intraprocedural CBCT imaging and tumor response in HCC is shown with the help of three dimensional quantification software [15]. Our comparable results showing strong correlations could also be drawn with such software.

Assessing lipiodol retention pattern by CBCT during the cTACE procedure Provides near real-time feed-back [14] before the patient leaves the angiography suite and enables the operator to set a more accurate endpoint and perform additional treatment if necessary.

This study has limitations such as small size of the study group, its retrospective design and lack of a control group. Due to the small size of our group, interpretation of CT and MRI studies obtained after cTACE and CBCT images are prone to observer bias. The one-month follow-up treatment response evaluation may be inferior to the more commonly practiced 2–3 month follow-up treatment response evaluation. This might have introduced an element of bias. Our results need to be validated further with larger and better designed studies.

## Conclusion

In conclusion, during cTACE for HCC tumors cone beam CT is more accurate in detection of lipiodol retention pattern compared to fluoroscopy. The pattern of lipiodol retention assessed by CBCT can serve as a prognostic indicator of short-term response and could be a reliable intraprocedural monitoring modility during cTACE.

## Abbreviations

CBCT: Cone beam computed tomography
CR: Complete response
CT: Computer tomography
cTACE: Conventional transarterial chemoembolization
EASL: European Association for the Study of the Liver
ECOG: Eastern Cooperative Oncology Group
HCC: Hepatocellular carcinoma
INR: International normalized ratio
MDCT: Multidetector computer tomography
mRECIST: Modified response evaluation criteria in solid tumors
MRI: Magnetic resonance imaging
PD: Progressive disease
PR: Partial response
SD: Stable disease
US: Ultrasonography
